# Regulation of IκBα expression involves both NF-κB and the MAP kinase signaling pathways

**DOI:** 10.1186/1476-9255-2-10

**Published:** 2005-10-05

**Authors:** Ning Zhang, Muhammad H Ahsan, Lingyun Zhu, Lidia C Sambucetti, Anthony F Purchio, David B West

**Affiliations:** 1 Xenogen Corporation, 860 Atlantic Avenue, Alameda, California 94501, USA

**Keywords:** IkappaB, NF-κB, MAP kinase, bortezomib, lipopolysaccharide, bioluminescent imaging

## Abstract

IκBα is an inhibitor of the nuclear transcription factor NF-κB. Binding of IκBα to NF-κB inactivates the transcriptional activity of NF-κB. Expression of IκBα itself is regulated by NF-κB, which provides auto-regulation of this signaling pathway. Here we present a mouse model for monitoring *in vivo *IκBα expression by imaging *I*κ*B*α-*luc *transgenic mice for IκBα promoter driven luciferase activity. We demonstrated a rapid and systemic induction of IκBα expression in the transgenic mice following treatment with LPS. The induction was high in liver, spleen, lung and intestine and lower in the kidney, heart and brain. The luciferase induction in the liver correlated with increased IκBα mRNA level. Pre-treatment with proteasome inhibitor bortezomib dramatically suppressed LPS-induced luciferase activity. The p38 kinase inhibitor SB203580 also showed moderate inhibition of LPS-induced luciferase activity. Analysis of IκBα mRNA in the liver tissue showed a surprising increase of the IκBα mRNA after bortezomib and SB203580 treatments, which could be due to increased IκBα mRNA stability. Our data demonstrate that regulation of IκBα expression involves both the NF-κB and the p38 signaling pathways. The *I*κ*B*α-*luc *transgenic mice are useful for analyzing IκBα expression and the NF-κB transcriptional activity *in vivo*.

## Introduction

IκBα is an inhibitor of nuclear transcription factor NF-κB, which regulates the expression of proinflammatory and cytotoxic genes [1]. In nonstimulated cells NF-κB proteins are present in the cytoplasm in association with specific inhibitors IκBα, IκBβ and IκBγ. Stimulation by extra-cellular inducers results in the phosphorylation and degradation of IκB through a ubiquitin-proteasome pathway, allowing NF-κB to translocate into the nucleus to activate the transcription of target genes [2,3]. The IκBα gene contains functional NF-κB sites in the promoter region. Transcriptional activation of IκBα expression by NF-κB leads to rapid re-synthesis of IκBα protein and blockade of NF-κB nuclear translocation [4,5]. This auto-regulatory loop is both sensitive to and rapidly influenced by NF-κB activating stimuli [6]. In addition, phosphorylation of IκB kinase and the activation of NF-κB also involve the MAP kinase signaling pathways [7].

In this paper we describe and characterize an *I*κ*B*α-*luc *transgenic mouse that was used for monitoring IκBα expression through bioluminescent imaging. We tested the effect of bortezomib and several MAP kinase inhibitors on LPS-induced IκBα expression. The results that follow suggest that, in addition to NF-κB, the MAP kinase signaling pathway is involved in controlling IκBα expression.

## Materials and methods

### Construction of pIκBα-luc vector and generation of *I*κ*B*α-*luc *transgenic mice

A mouse BAC clone containing the mouse IκBα gene was isolated from a CT7 mouse BAC library (Invitrogen, Carlsbad, CA). A 11.0 kb promoter fragment containing sequences 5' to the first ATG for the mouse IκBα gene was obtained by the *RED *cloning method [8] and cloned upstream of the firefly luciferase gene in the pGL3-Basic vector (Promega, Madison, WI). A 0.8 kb human β-globin intron 2 was placed between the IκBα promoter and the luciferase gene to optimize the luciferase expression in transgenic mice. The transgene cassette was separated from the vector backbone sequences and used for pronuclear injection into Balb/C mouse strain embryos. These steps yielded the transgenic model henceforth designated Balb/C-Tg(*I*κ*B*α-*luc*)Xen and abbreviated in the text as *I*κ*B*α-*luc*.

### Reagents

We purchased bacterial lipopolysaccharide (LPS, from *Salmonella *abortus equi), PD098580 from Sigma-Aldrich Chemical Co., (St. Louis, MO), Bortezomib (VALCADE, PS-341) from Millennium Pharmaceuticals, Inc. (Cambridge, MA), SB203580 from EMD Biosciences, Inc. (La Jolla, CA) and SP600125 from A.G. Scientific, Inc. (San Diego, CA).

### *In vivo *imaging of luciferase activity

*In vivo *imaging was performed using an IVIS^® ^Imaging System 100 Series (Xenogen Corp., Alameda, CA). *I*κ*B*α-*luc *transgenic mice were anesthetized with isoflurane and injected intraperitoneally with 150 mg/kg of luciferin (Biosynth, A.G., Switzerland). Ten minutes after the luciferin injection, mice were imaged for 1–10 seconds. Photons emitted from specific regions were quantified using Living Image^® ^software (Xenogen Corp.). *In vivo *luciferase activity is expressed as photons/second/cm^2^.

### Study of *in vivo IκBα *gene regulation using *I*κ*B*α-*luc *transgenic mice

*I*κ*B*α-*luc *transgenic mice of 3–6 months of age were injected with LPS (1 mg/kg, *i.p*.). Control mice were injected with saline. At selected time points, mice were imaged for the luciferase signal. To test the effect of various compounds, mice were pre-treated with bortezomib (1 mg/kg, *i.v*.), PD098059 (10 mg/kg, *i.v.*), SP600125 (20 mg/kg, *i.v.*), or SB203580 (5 mg/kg, *i.v.*) 1 hour prior to the LPS injection.

### Tissue luciferase activity

Selected organs were removed and homogenized in 3 volumes of PBS containing a protease inhibitor cocktail (Roche Applied Science, Indianapolis, IN) and lysed with passive lysis buffer (Promega). After centrifugation at 14,000-rpm for 10 min at 4°C, the supernatant was collected. Luciferase activity was assayed using the Luciferase Assay System (Promega) and a Turner Design, TD 20/20, Luminometer (Sunnyvale, CA). Protein concentration was estimated with Bradford reagent (Sigma-Aldrich).

### Northern blot analysis

Total RNA was isolated from mouse tissue using RNAwiz (Ambion, Austin, TX) and further purified using the RNAeasy kit (Qiagen Inc., Valencia, CA). A total of 2 μg of RNA sample was analyzed by Northern blot using a NorthernMax system (Ambion). A 482 nt IκBα cDNA fragment was amplified (forward primer: 5'- GCTCTAGAGCAATCATCCACGAAGAGAAGC-3'; reverse primer: 5'- CGGAATTCGCCCCACATTTCAACAAGAGC-3') and cloned into the pBlueScript SK vector (Stratagene, La Jolla, CA) that was linearized with *XbaI *and *EcoRI*. Single strand antisense IκBα RNA probe was prepared by transcription with T7 polymerase using a Strip-EZ kit (Ambion). After hybridization, the signal was detected using a BrightStar BioDetect kit (Ambion)

### Statistics

Nonparametric tests for significance were used to test whether changes in luciferase signal from baseline were significantly greater than zero within groups (sign test) and whether the changes from baseline were significantly different between treatment groups (Mann-Whitney test). Values are presented as means ± one standard error in the graphs and text unless otherwise noted. For some statistical tests genders were combined to increase sample number in each group. All significance levels are two-sided.

## Results

### Induction of *I*κ*B*α expression by LPS

**Figure 1:**
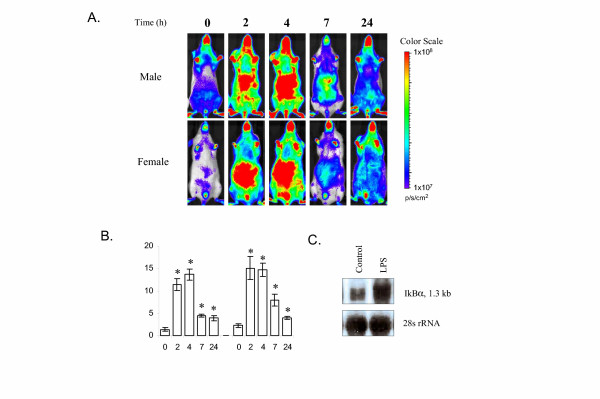
Imaging analysis of luciferase expression in *I*κ*B*α-*luc *transgenic mice treated with LPS. **A. ***I*κ*B*α-*luc *transgenic mice were imaged at T = 0, 2, 4, 7 and 24 hours after treatment with LPS (1 mg/kg, *i.p*., n = 4 for males, n = 6 for females). Representative mice from each treatment group are shown. The color overlay on the image represents the photons/second emitted from the mouse body in accord with the pseudo-color scale shown on the right of the images. Red represents the highest photons/sec while blue represents the lowest photons/sec. **B. **Quantification of the luciferase signal from the abdominal region of the body. Data are means luciferase activity (billion photon/second) ± SE. Statistical analysis was done for male and female combined data. * indicates a significant induction of luciferase signal by LPS (P = 0.002). **C. **Northern blot analysis of IκBα mRNA in the liver tissue. Liver tissue was harvested from saline (control) or LPS treated *I*κ*B*α-*luc *female mice at 4 hours after treatment and processed for RNA isolation. A total of 2 μg of RNA was analyzed by Northern blot. Equal loading was demonstrated by 28S rRNA.

We generated *I*κ*B*α-*luc *transgenic mice and screened for their response to LPS treatment through bioluminescent imaging of luciferase activity. Transgenic mice from all founder lines showed inducible luciferase expression after LPS treatment. One transgenic line was selected for this study. In untreated *I*κ*B*α-*luc *mice, basal luciferase signal was detected throughout the entire body. Male and female mice showed similar levels of basal luciferase signal. After LPS treatment, an induction of luciferase signal was observed at 2 hours after treatment. The signal remained highly induced at 4 hours and started to decline at 7 hours. By 24 hours, the signal declined to near baseline levels (Figure 1A). Anatomically, the induction was higher in hepatic and intestinal regions of the abdomen than that in other parts of the body.

Luciferase signals from the abdominal region of LPS-treated mice were quantified using the Living Image^® ^software to produce the data shown in Figure 1B. At the peak of induction 2 to 4 hours after injection, the luciferase signals were increased 6 to 10-fold by LPS as compared with basal luciferase signal at T = 0 hour. At 24 hours, the luciferase signal was still 2 to 3-fold greater than basal levels.

### IκBα expression is induced in multiple tissues after LPS treatment

Table 1 displays the luciferase activity in selected organs in *I*κ*B*α-*luc *mice. In untreated mice, *ex vivo *luciferase activity was detected in all the dissected organs of both sexes. The pattern of luciferase expression of the male tissues was similar to that of the female tissues. The luciferase activity was the highest in liver, spleen and lung, lowest in heart, and intermediate in intestine, kidney and brain. In LPS treated mice, all the examined organs showed a significant induction of the luciferase activity. Liver, spleen, lung and intestine showed dramatically higher luciferase expression than that in kidney, heart and brain. As calculated from the mean of the control mice, LPS treatment caused 19-to 23-fold luciferase induction in the liver, 19- to 28-fold in the spleen, 8-fold in the lung, 19- to 52-fold in the intestine, 6-to 11-fold in the kidney, 54- to 63-fold in the heart, 5- to 7-fold in the brain.

**Table 1:** *Ex vivo *measurement of luciferase activity (Unit/μg protein). Selected organs were harvested from *IkBα-luc *mice that were untreated (control, n = 3) or treated with LPS (1 mg/kg, *i.p*., n = 3) at 4 hours prior to the harvesting.

	**Mean ± SE**			
	
	**MALE**		**FEMALE**	
**ORGANS**	**Control**	**LPS**	**Control**	**LPS**

**Liver**	157 ± 30	3651 ± 48*§	157 ± 2	2933 ± 69*
**Spleen**	363 ± 69	6906 ± 878*	218 ± 58	6203 ± 1414*
**Lungs**	430 ± 112	3549 ± 291*	348 ± 52	2718 ± 452*
**Intestine**	89 ± 39	4640 ± 601*	73 ± 9	1367 ± 598*
**Kidney**	65 ± 7	709 ± 62*	67 ± 5	414 ± 26*
**Heart**	15 ± 2§	951 ± 141*	7 ± 2	405 ± 8*
**Brain**	72 ± 13	513 ± 84*	73 ± 9	384 ± 52*

*Difference from controls significant at P ≤ 0.05 by Mann-Whitney nonparametric test.

We further attempted to establish a correlation between luciferase activity and IκBα mRNA expression. In the liver tissue of un-treated mice, IκBα mRNA expression was detectable. Following LPS treatment, an induction of IκBα mRNA expression was observed (Figure 1C), which correlated with the increase of luciferase activity in the liver.

### Bortezomib inhibited LPS-induced IκBα expression

**Figure 2:**
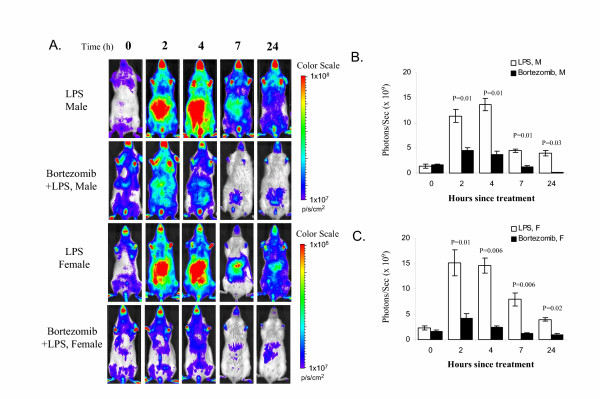
Effect of bortezomib on LPS-induced luciferase expression. **A. ***I*κ*B*α-*luc *transgenic mice were pre-treated with bortezomib (1 mg/kg, *i.v. *n = 5) at 1 hour prior to the LPS treatment. The positive control mice (n = 4 for males, n = 6 for females) were pre-injected with saline. All the mice were imaged at T = 0, 2, 4, 7 and 24 hours after the LPS treatment. **B, C. **Quantification of the luciferase signal from the abdominal region of the body for male and female mice respectively. Data are expressed as billion photons/second. Nonparametric significance levels for the difference between treatment groups were determined by a Mann-Whitney test and are presented above the bars.

Using the *I*κ*B*α-*luc *model, we tested the effect of bortezomib on LPS-induced IκBα expression *in vivo*. As shown in Figure 2A, pre-treatment of the *I*κ*B*α-*luc *mice with bortezomib significantly inhibited LPS-induced luciferase expression in the whole body, especially in liver and intestine where the luciferase signal was highly induced. Quantification of the luciferase signal showed that inhibition of luciferase activity by bortezomib was significant at all the time points in both male and female mice (Figure 2B, C). At the peak of induction at 2–4 hours, bortezomib inhibited 70–80% of LPS-induced luciferase activity in the abdominal region.

### Bortezomib inhibited LPS-induced *I*κ*B*α expression in all the organs except the brain

**Figure 3:**
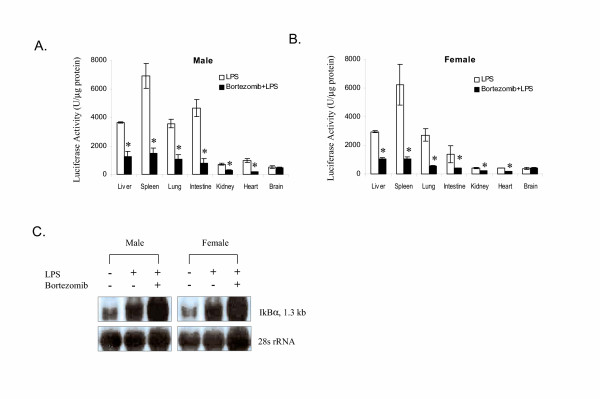
Effect of bortezomib pre-treatment on the LPS-induced luciferase activity in selected tissues in *I*κ*B*α-*luc *male (**A**) and female (**B**) mice (n = 3 for both genders). Mice were injected with bortezomib (1 mg/kg, *i.v*.) 1 hour prior to the LPS treatment (1 mg/kg, *i.p*.). Mice treated with LPS alone were used as positive controls. Organs were harvested from all the mice at 3 hours after the LPS injection and processed for luciferase activity.* indicates a significant reduction in signal by bortezomib (P = 0.05). **C. **Northern blot analysis of IκBα mRNA in the liver tissue. *I*κ*B*α-*luc *transgenic mice were sacrificed at 3 hours after LPS injection. Liver tissue was harvested and processed for RNA isolation. A total of 2 μg of RNA was analyzed by Northern blot. Equal loading was demonstrated by 28S rRNA.

We examined the effect of bortezomib on LPS-induced IκBα expression in selected organs (Figure 3A, B). In comparison to the LPS-treated mice, mice pre-treated with bortezomib showed significant inhibition of luciferase induction in all organs examined except the brain. The inhibition ranges from 50% to 80% in examined tissues excluding the brain.

We further examined the effect of bortezomib on IκBα mRNA induction by LPS. In both male and female mice, pre-treatment with bortezomib increased LPS-induced IκBα mRNA level in the liver tissue (Figure 3C).

### Effect of the MAP kinase inhibitors on IκBα induction by LPS

**Figure 4:**
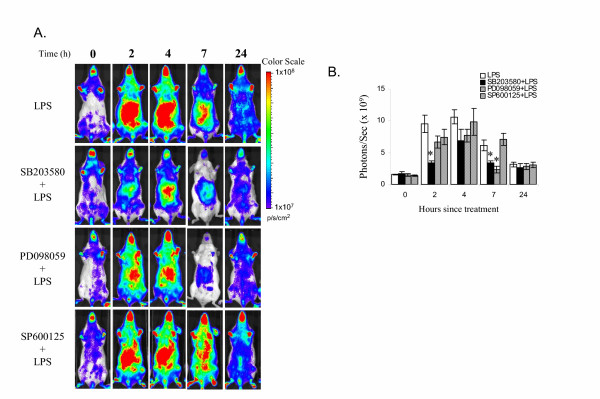
Effect of MAP kinase inhibitors on LPS-induced luciferase expression. **A. **Female *I*κ*B*α-*luc *transgenic mice were pre-treated with SB203580 (5 mg/kg, *i.v.*, n = 5), PD098059 (10 mg/kg, *i.v.*, n = 5), or SP600125 (20 mg/kg, *i.v.*, n = 8) at 1 hour prior to the LPS treatment. The positive control mice were pre-injected with DMSO (n = 8). All the mice were imaged at T = 0, 2, 4, 7 and 24 hours after LPS treatment. Representative mice are shown for each group. **B. **Quantification of the luciferase signal from liver region and the data were expressed as photons/second/cm^2^.

We examined the effect of MAP kinase inhibitors SB203580, PD098059 and SP600125 on LPS-induced IκBα expression. The bioluminescent images and the quantification are presented in Figure 4A and 4B respectively. Pre-treatment of the *I*κ*B*α-*luc *mice with SB203580 moderately inhibited LPS-induced luciferase expression. PD098059 pre-treated mice also had lower luciferase activity as compared to the LPS-treated positive control mice. However, the difference was significant at 7 hours only (Figure 4B). SP600125 failed to affect LPS-induced luciferase expression.

**Figure 5:**
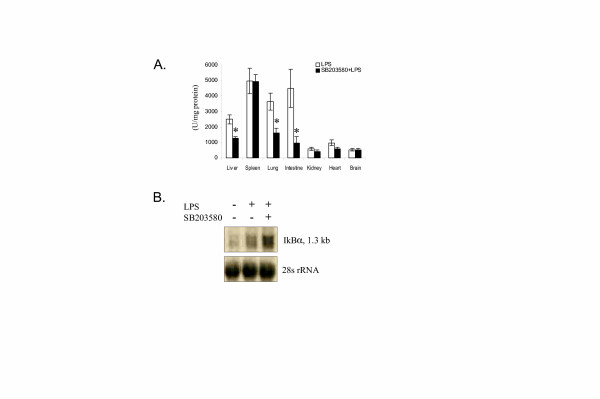
*Ex vivo *measurement of the effect of SB203580 on LPS-induced luciferase expression. **A. **Selected organs were harvested from SB203580 pre-treated mice and LPS treated control mice at 4 hours after the LPS injection. * indicates a significant difference between vehicle (DMSO) + LPS and SB203580 + LPS (p = 0.05; sign test). **B. **Northern blot analysis of IκBα mRNA in the liver tissue. *I*κ*B*α-*luc *transgenic mice were sacrificed at 3 hours after LPS injection. Liver tissue was harvested and processed for RNA isolation. A total of 2 μg of RNA was analyzed by Northern blot. Equal loading was demonstrated by 28S rRNA.

We further analyzed the luciferase activity in selected organs harvested from SB203580-pre-treated mice at 3 hours after the LPS injection. As shown in Figure 5A, SB203580 significantly inhibited LPS-induced luciferase activity in liver, lung, and intestine, but not in the spleen, brain, kidney or heart.

The effect of SB203580 on IκBα mRNA induction by LPS is shown in Figure 5B. Pre-treatment with SB203580 increased LPS-induced IκBα mRNA level in the liver tissue of the *I*κ*B*α-*luc *mice.

## Discussion

The mouse IκBα promoter contains 6 putative NF-κB binding sites that mediate the NF-κB regulation [9]. Induction of *I*κ*B*α-*luc *expression in the early stage of the LPS response is consistent with a tight auto-regulation of the NF-κB signaling pathway by IκBα [6]. By reflecting NF-κB transcriptional activity, the luciferase signal in the *I*κ*B*α-*luc *mouse provides a convenient approach for *in vivo *monitoring of NF-κB activation.

It has been shown previously that LPS treatment causes degradation of IκBα protein within 40 minutes, followed by induction of IκBα mRNA that results in rapid recovery of the IκBα protein by 3 hours. As a result, maximal NF-κB activation occurred 1 hour after LPS treatment but started to decline at 3–6 hours post treatment [10]. In agreement, our *in vivo *imaging data demonstrated an induction of luciferase activity at 2 to 4 hours after treating the *I*κ*B*α-*luc *mice with LPS, followed by decline of the luciferase activity at 7 and 24 hours. In addition, we also observed a slight gender difference of the kinetics of NF-κB activation following LPS treatment. Male mice showed a peak of induction at 4 hours, followed by a sharp decrease at 7 hours. Female mice showed a peak of induction at 2 hours, followed by a sequential decrease at 7 and 24 hours. This indicates that LPS-induced inflammation process may be sustained longer in female mice than in male mice.

*Ex vivo *analysis of selected tissues of *I*κ*B*α-*luc *mice showed baseline luciferase expression in liver, spleen and lung, with lower expression in intestine, kidney, heart and brain. Significant induction of luciferase expression was observed in all of these organs in both male and female mice after LPS treatment, with higher luciferase activity observed in liver, spleen and intestine as compared to other tissues (Table 1). This is consistent with the bioluminescent imaging analysis of luciferase activity in the live mice that shows higher luciferase signals were present in both hepatic and intestinal regions than other parts of the body (Figure 1A). High extent of luciferase induction in the liver, spleen, lung and intestine by LPS is consistent with IκBα degradation and NFκB activation in these organs in response to endotoxemia [11-13]. When male and female mice are compared, the luciferase signal in intestine was significantly higher in the LPS-treated male mice as compared with the female mice. The difference could be due to the difference of the kinetics of luciferase induction between male and female mice or simply due to a relatively small sample number used for this study.

Bortezomib inhibited LPS-induced luciferase activity by 70–80% in the *I*κ*B*α-*luc *mice, which is confirmed by a broad suppression of luciferase activity in all the analyzed tissues except the brain. Bortezomib is an inhibitor of proteasome activity that is required for IκB degradation and subsequent nuclear translocation of NF-κB [14]. In addition, bortezomib can also inhibit other cell signaling pathways, such as mitogen-activated protein kinase growth signaling, causing inhibition of cell proliferation and induction of cell apoptosis [15,16]. Analysis of the IκBα mRNA showed that bortezomib pre-treatment caused a further increase of LPS-induced IκBα mRNA in the liver. Since the transcriptional activity of the IκBα promoter was suppressed bortezomib, we suspect that the increase of IκBα mRNA after bortezomib treatment should be due to an increase of IκBα mRNA stability. These data suggest that inhibition of NF-κB mediated inflammation by bortezomib may be due to a broad range of effects, affecting processes such as IκB protein degradation and IκBα mRNA stability.

Several MAP kinase inhibitors were tested for their effect on LPS-induced NF-κB activation. We demonstrated that pre-treatment with p38 MAP kinase inhibitor SB203580 at a dose of 5 mg/kg partially inhibited LPS-induced luciferase expression in the *I*κ*B*α-*luc *mice in liver, lung and intestine. It has been reported that SB203580 inhibits inflammatory cytokine production *in vivo *in both mice and rat with IC50 value of 15 to 25 mg/kg [17]. In another report, it was shown that SB203580 at 5, 10 and 20 mg/kg produced a dose dependent inhibition on TNF-alpha production *in vivo *[18]. Therefore, it is likely that the SB203580 dose used in our study had an inhibitory effect on p38 MAP kinase activation. We also showed that the ERK MAP kinase inhibitor PD098059 at 10 mg/kg partially inhibited LPS-induced luciferase expression at 7 hours. At this dose, PD098059 was able to suppress ERK1/2 phosphorylation *in vivo *[19]. We further showed that JNK kinase inhibitor SP600125 at 20 mg/kg had no effect on LPS-induced luciferase expression. At this dose, SAPK/JNK MAP kinase phosphorylation can be totally inhibited in the liver tissue [20].

In summary, we have produced a transgenic mouse in which luciferase expression is driven by the IκBα promoter. We observed a ubiquitous expression and induction of IκBα in the *I*κ*B*α-*luc *transgenic mice by LPS. We demonstrated involvement of both the NF-κB and the p38 MAP kinase signaling pathways in the induction of IκBα expression by LPS.

Clinically, NF-κB activation is involved in many chronic disease conditions, such as rheumatoid arthritis, atheroscleorosis, asthma and tumor development [21,22]. The luciferase activity in the *I*κ*B*α-*luc *mice could be used as a sensor for monitoring the NF-κB activation and to further understand how NF-κB activation contributes to the initiation and progression of these disease conditions. In addition, *I*κ*B*α-*luc *mice could also be used for testing or even screening of novel NF-κB inhibitors for therapeutic potential.
